# 

**Published:** 1862-10

**Authors:** 


### CONTENTS.

ORIGINAL ESSAYS AND COMMUNICATIONS.

Integrity By Dr. W. H. Atkinson ........ 437
Mechanical Dentistry. By B. W. Franklin, of New York City ........ 440
Doctrines on Exposed Pulps By Dr. W. H. Atkinson ........ 445
Hints on Vulcanite Work. By Geo. E. Snow ........ 478

SELECTIONS.

Dentition and its Derangements. Lecture X, Part 1 ........ 447
Dentition and its Derangements. Lecture X, Part 2 ........ 450
Local Anæsthesia ........ 455
Spasms from Intestinal Irritation and Teething ........ 456
A New Splint for Fractures of the Jaw ........ 458
A Portable Styptic ........ 459
Keroselene as an Anæsthetic ........ 460
Logwood as an Antiseptic ........ 461
New and Cheap Method of Ventilation, applicable to any Apartment ........ 262
Curiosities of Leech Culture ........ 466
Facial Paralysis ........ 467
Phosphorus Necrosis ........ 468
Dialysis ........ 471
London Surgeons ........ 477

EDITORIAL.

American Dental Convention ........ 480
Plastic Metallic Fillings ........ 481
Logwood as an Antiseptic ........ 481
The Dental Cosmos ........ 482
A New Drill Stock ........ 482
Local Dental Societies ........ 483
Removal ........ 483
The Dental Quarterly ........ 484
Promotion ........ 484
Fully Prepared ........ 484
Lovering's Etherial Varnish ........ 484

